# Supplementary material for: Helminth exposure protects against murine SARS-CoV-2 infection through macrophage dependent T cell activation
Source: bioRxiv. 2022 Nov 10:2022.11.09.515832. Preprint. [Version 1] doi: 10.1101/2022.11.09.515832 (PMC9665339; doi:10.1101/2022.11.09.515832)
Supplement: 1 [file NIHPP2022.11.09.515832V1-supplement-1.pdf]

Figure S1

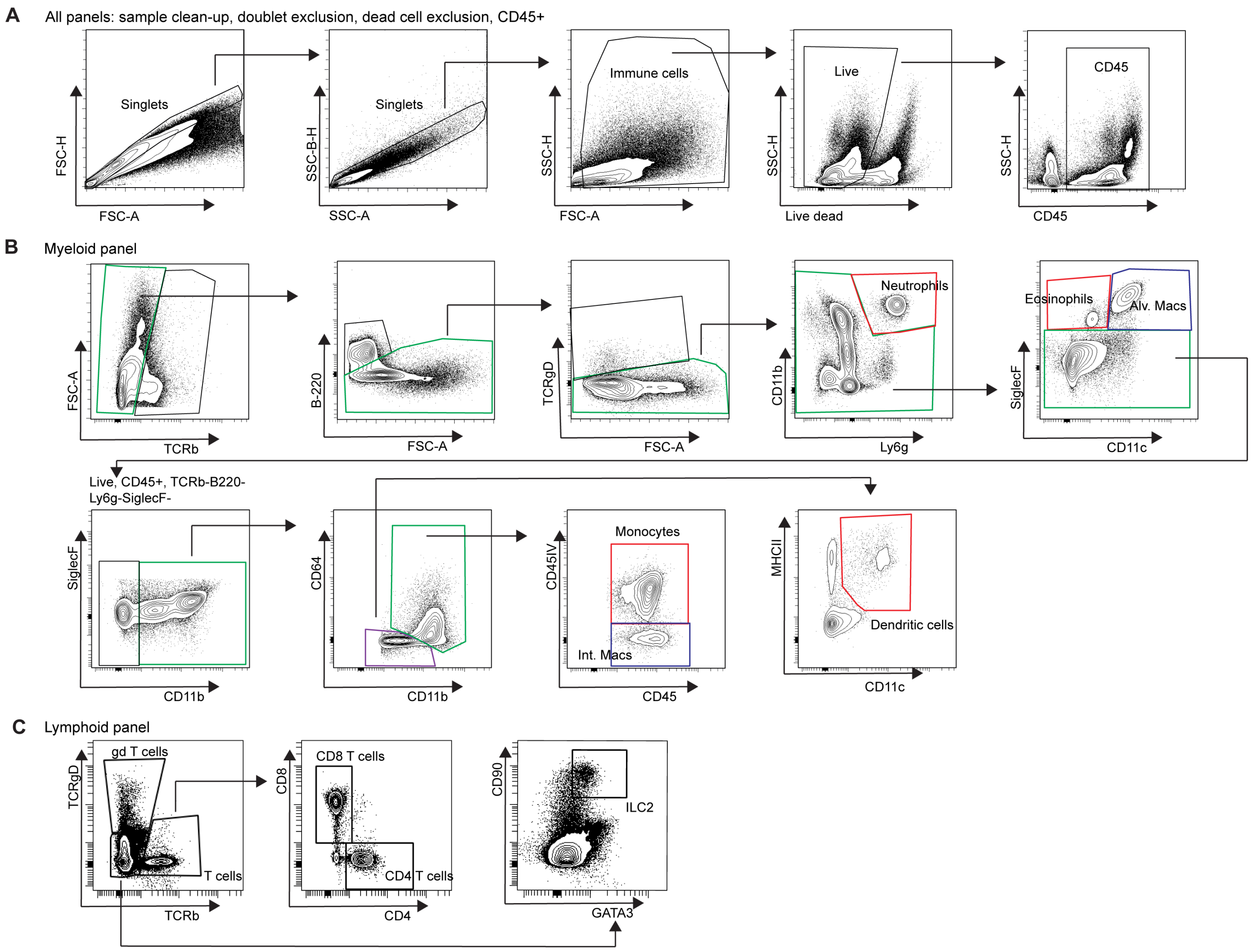

A
All panels: sample clean-up, doublet exclusion, dead cell exclusion, CD45+
Singlets
FSC-H
FSC-A
Singlets
SSC-B-H
SSC-A
Immune cells
SSC-H
FSC-A
Live
SSC-H
Live dead
CD45
SSC-H
CD45
B
Myeloid panel
FSC-A
TCRb
B-220
FSC-A
TCRgD
FSC-A
Neutrophils
CD11b
Ly6g
Eosinophils
Alv. Macs
SiglecF
CD11c
Live, CD45+, TCRb-B220-
Ly6g-SiglecF-
SiglecF
CD11b
CD64
CD11b
Monocytes
CD45IV
Int. Macs
CD45
MHCII
Dendritic cells
CD11c
C
Lymphoid panel
gd T cells
TCRgD
T cells
TCRb
CD8 T cells
CD8
CD4 T cells
CD4
CD90
ILC2
GATA3

Supplementary Figure 1: Gating strategies to identify immune cell subsets by spectral cytometry.

Single cell suspensions were prepared from the lungs of animals i.v. injected with a fluorescently labeled panCD45 antibody 3 min before euthanasia to allow for identification of cells located within the pulmonary vasculature vs. the cells in the lung interstitium or airways. Strategies employed for sample clean-up (**A**), identifying myeloid populations (**B**) and identifying lymphoid populations (**C**).

Figure S2

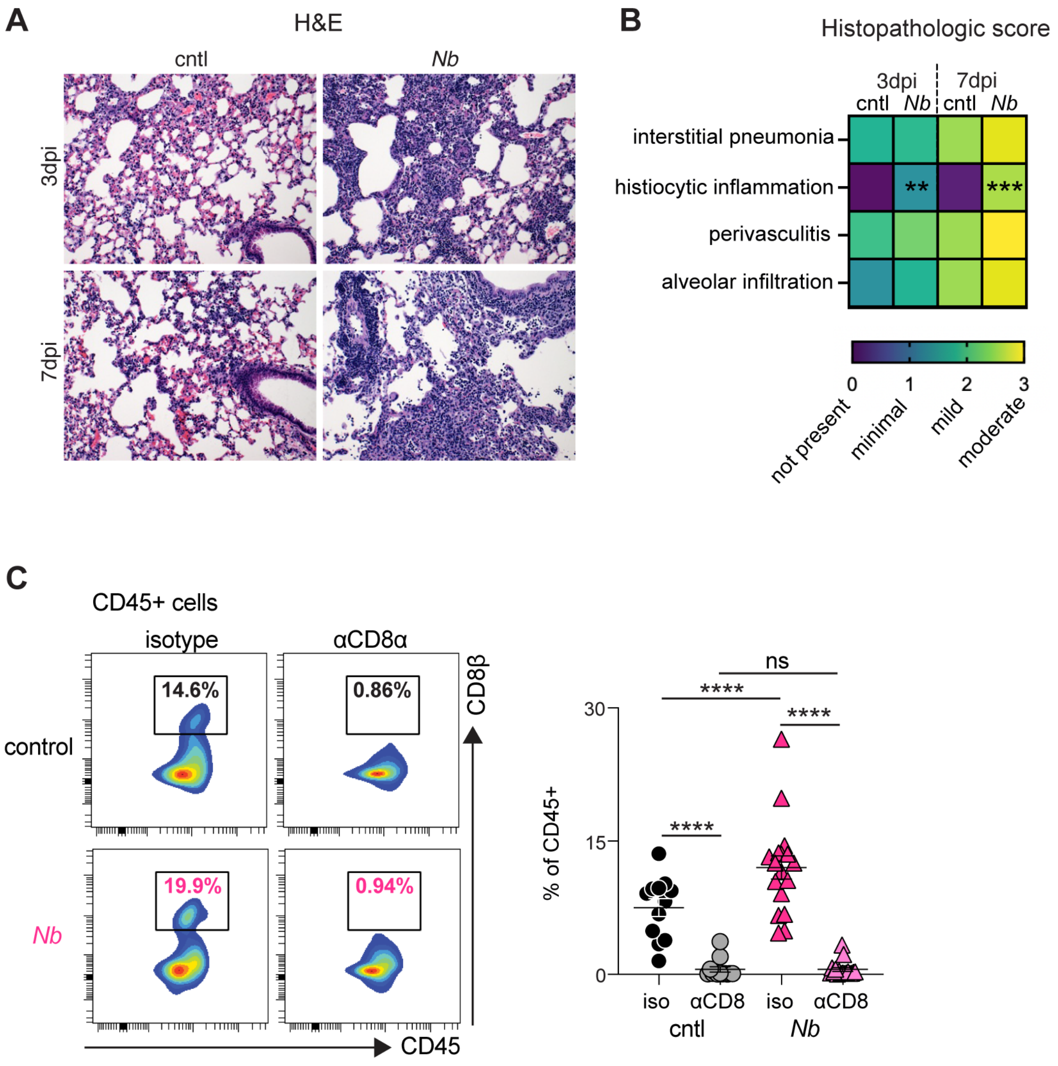
A
H&E
cntl
Nb
3dpi
7dpi
B
Histopathologic score
3dpi
7dpi
cntl
Nb
cntl
Nb
interstitial pneumonia
histiocytic inflammation
perivasculitis
alveolar infiltration
**
***
0
1
2
3
not present
minimal
mild
moderate
C
CD45+ cells
isotype
αCD8α
control
Nb
14.6%
0.86%
19.9%
0.94%
CD8β
CD45
% of CD45+
30
15
0
ns
****
****
****
iso
αCD8
iso
αCD8
cntl
Nb

Supplementary Figure 2: Previous *N. brasiliensis* infection drives lymphocytic inflammation following SCV2 challenge.

(**A-B**) K18-hACE2 mice were infected with 500 L3 *N. brasiliensis* (*Nb*) larvae s.c. or left uninfected. After 28 days, animals were challenged i.n. with 10^3 TCID50 SCV2 and lungs harvested at 3 or 7d post SCV2 for histopathological analysis. *n*=7-10 mice/group; 2 independent experiments. (**A**) Representative hematoxylin and eosin (H&E) stained lung tissue sections. (**B**) Heat map representation of histopathological scores as determined by a board-certified veterinary pathologist. Statistical significance between different groups at different time point was determined using an unpaired Student's *t*-test with Graph-Pad Prism software. (**C**) K18-hACE2 mice were inoculated with 500 *Nb* larvae by s.c. injection at d-28. Mice were then treated with either anti-CD8α or rat IgG2b isotype control on d-5, d-3, d-1 prior to SCV2 challenge on d0. Lung tissue was harvested at 7dpi to determine frequency of CD8+ T cells by flow cytometry using a CD8β antibody. *n*=11-17 mice/group; 2 independent experiments. Statistical significance was assessed using a linear mixed-effects model with pairwise comparison using JMP software. ns $p>0.05$; * $p<0.05$; ** $p<0.01$; *** $p<0.001$; **** $p<0.0001$

Figure S3

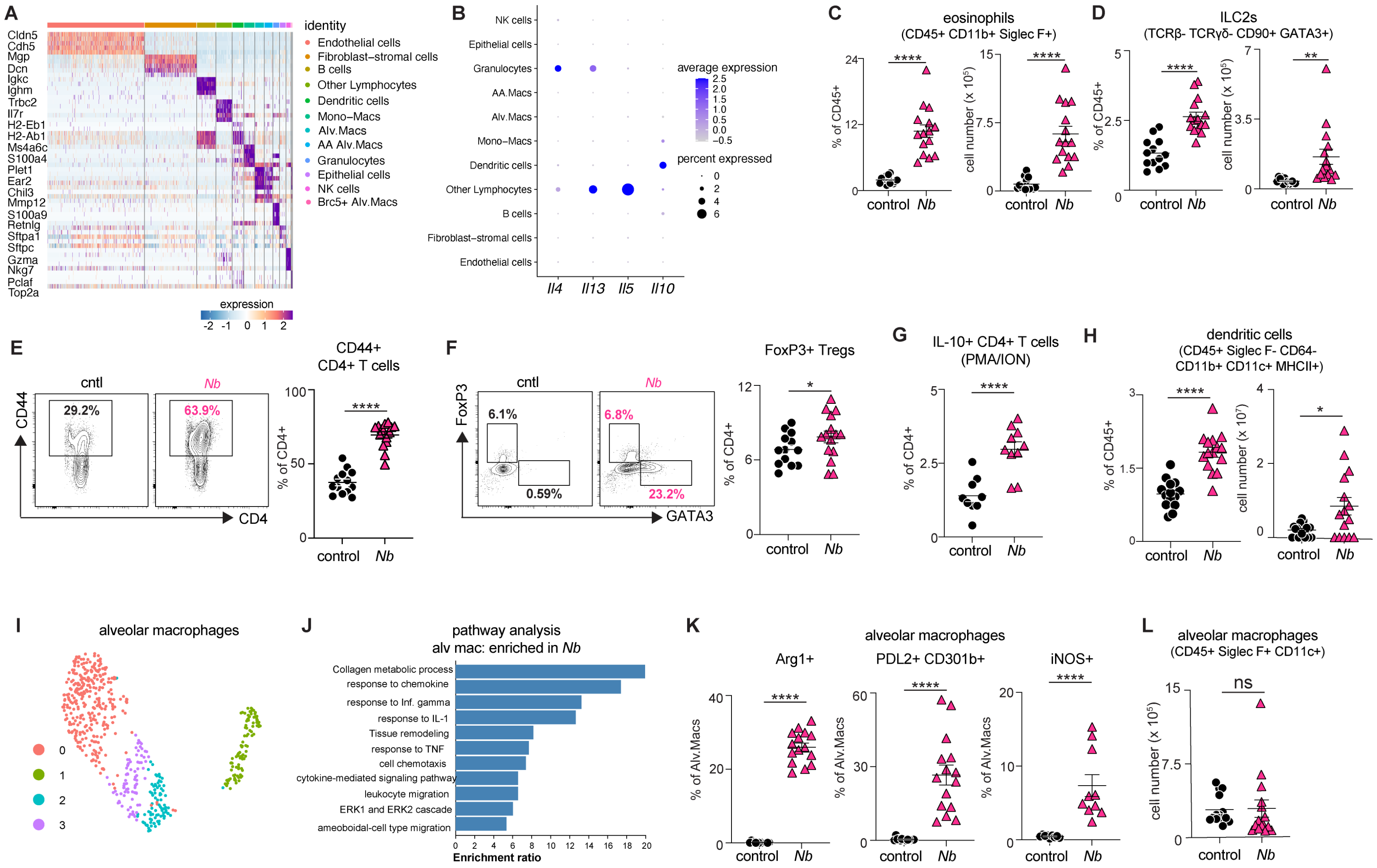
A
identity
Endothelial cells
Fibroblast–stromal cells
B cells
Other Lymphocytes
Dendritic cells
Mono–Macs
Alv.Macs
AA Alv.Macs
Granulocytes
Epithelial cells
NK cells
Brc5+ Alv.Macs
expression
B
average expression
percent expressed
Il4 Il13 Il5 Il10
C
eosinophils
(CD45+ CD11b+ Siglec F+)
D
ILC2s
(TCRβ- TCRγδ- CD90+ GATA3+)
E
CD44+
CD4+ T cells
F
FoxP3+ Tregs
G
IL-10+ CD4+ T cells
(PMA/ION)
H
dendritic cells
(CD45+ Siglec F- CD64-
CD11b+ CD11c+ MHCII+)
I
alveolar macrophages
J
pathway analysis
alv mac: enriched in Nb
Enrichment ratio
K
alveolar macrophages
Arg1+
PDL2+ CD301b+
iNOS+
L
alveolar macrophages
(CD45+ Siglec F+ CD11c+)

Supplementary Figure 3: Previous *N. brasiliensis* infection skews the lung micro-environment towards a Type 2 and regulatory phenotype.

K18-hACE2 mice were infected with 500 L3 *N. brasiliensis* (*Nb*) larvae s.c. or left uninfected. After 28 days, lungs were harvested and processed for scRNAseq (*n*=pool of 4-5 mice/group), flow cytometric analysis or multiplex cytokine assay (*n*=14-15 mice/group; 3 independent experiments). (**A**) Heat map depicting cluster defining genes used for cell type calling in Fig3A. (**B**) Normalized expression of *Il4*, *Il5*, *Il13* and *Il10* transcripts for each cell type. (**C-K**) Flow cytometric determination of (**C**) the frequency and number of $CD11b^+$ Siglec F^+ eosinophils, (**D**) the frequency and number of TCR^- $CD90^+$ $GATA3^+$ ILC2s, (**E**) frequency of $CD44^+$ $CD4^+$ T cells, (**F**) frequency of $FoxP3^+$ $CD4^+$ Tregs, (**G**) frequency of IL-10^+ $CD4^+$ T cells, (**H**) frequency and number of $CD64^-$ $CD11c^+$ $MHCII^+$ $CD11b^+$ dendritic cells. Statistical significance was assessed using a linear mixed-effects model with pairwise comparison using JMP software. (**I**) UMAP visualization of Seurat clustering of alveolar macrophages. (**J**) Pathway analysis showing enrichment in *Nb* alveolar macrophages compared to control. (**K**) Flow cytometric assessment of Arg1, PDL2, CD301b and iNOS expression by alveolar macrophages. (**L**) Number of alveolar macrophages as determined by flow cytometry. Statistical significance was assessed using a linear mixed-effects model with pairwise comparison using JMP software. Data are displayed as mean ± SEM. ns $p>0.05$; * $p<0.05$; ** $p<0.01$; *** $p<0.001$; **** $p<0.0001$

Figure S4

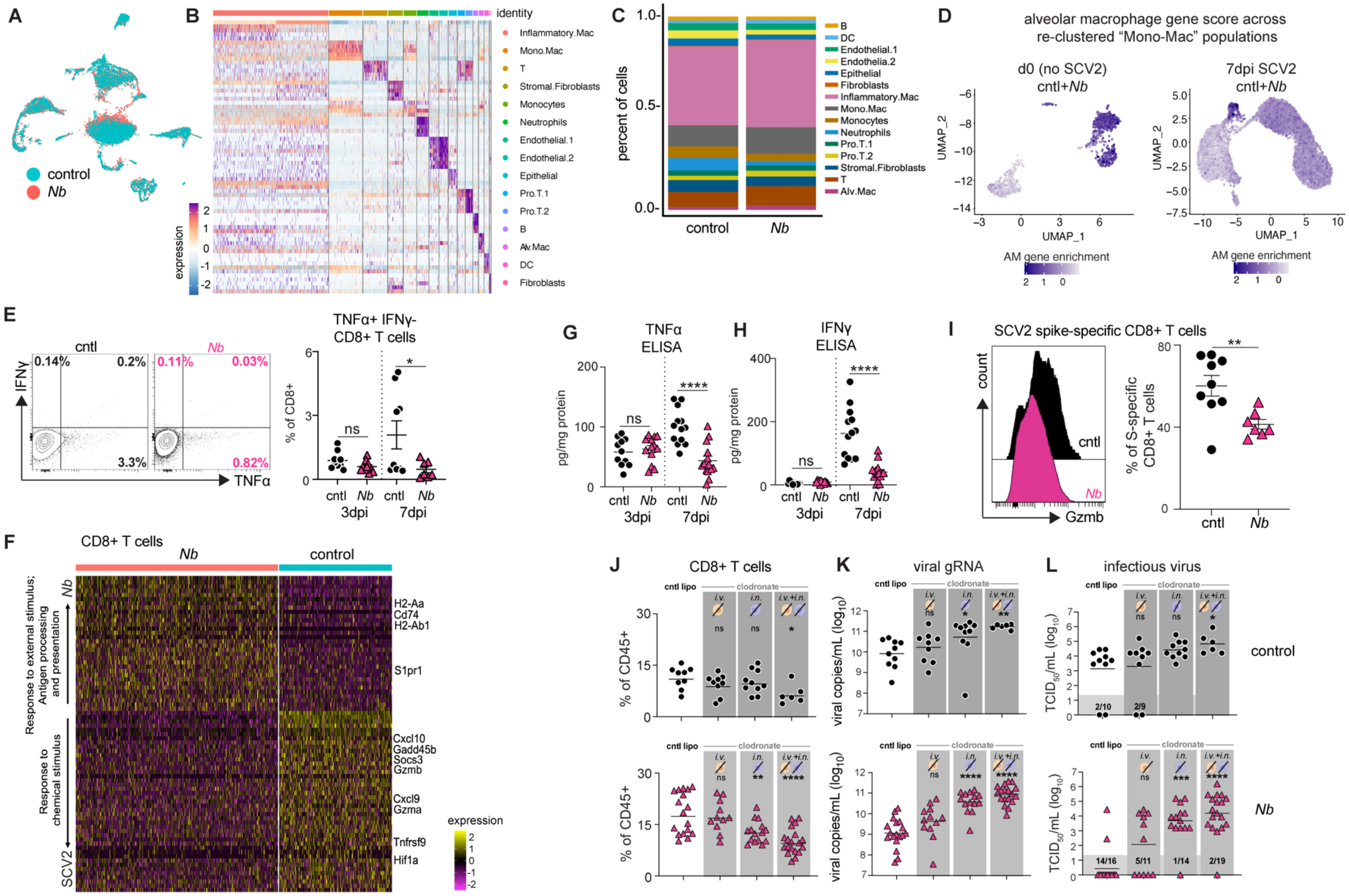

A
control
Nb
B
identity
Inflammatory.Mac
Mono.Mac
T
Stromal.Fibroblasts
Monocytes
Neutrophils
Endothelial.1
Endothelial.2
Epithelial
Pro.T.1
Pro.T.2
B
Alv.Mac
DC
Fibroblasts
expression
C
percent of cells
control
Nb
B
DC
Endothelial.1
Endothelia.2
Epithelial
Fibroblasts
Inflammatory.Mac
Mono.Mac
Monocytes
Neutrophils
Pro.T.1
Pro.T.2
Stromal.Fibroblasts
T
Alv.Mac
D
alveolar macrophage gene score across re-clustered "Mono-Mac" populations
d0 (no SCV2) cntl+Nb
7dpi SCV2 cntl+Nb
UMAP_1
UMAP_2
AM gene enrichment
E
cntl
Nb
IFNγ
TNFα
0.14%
0.2%
3.3%
0.11%
0.03%
0.82%
TNFα+ IFNγ- CD8+ T cells
% of CD8+
ns
*
cntl Nb cntl Nb
3dpi 7dpi
G
TNFα ELISA
pg/mg protein
ns
****
cntl Nb cntl Nb
3dpi 7dpi
H
IFNγ ELISA
pg/mg protein
ns
****
cntl Nb cntl Nb
3dpi 7dpi
I
SCV2 spike-specific CD8+ T cells
count
cntl
Nb
Gzmb
% of S-specific CD8+ T cells
**
cntl Nb
F
CD8+ T cells
Nb
control
Response to external stimulus; Antigen processing and presentation
Response to chemical stimulus
Nb
SCV2
H2-Aa
Cd74
H2-Ab1
S1pr1
Cxcl10
Gadd45b
Socs3
Gzmb
Cxcl9
Gzma
Tnfrsf9
Hif1a
expression
J
CD8+ T cells
cntl lipo
clodronate
i.v.
i.n.
i.v.+i.n.
% of CD45+
ns
ns
*
**
****
K
viral gRNA
viral copies/mL (log10)
ns
*
**
ns
****
****
L
infectious virus
TCID50/mL (log10)
ns
ns
*
ns
***
****
2/10
2/9
14/16
5/11
1/14
2/19
control
Nb

Supplementary Figure 4: Previous *N. brasiliensis* infection alters the pulmonary macrophage profile and pro-inflammatory cytokine responses after SCV2 challenge

(**A-I**) K18-hACE2 mice were infected with 500 L3 *N. brasiliensis* (*Nb*) larvae s.c. or left uninfected (control). After 28 days, animals were challenged i.n. with 10^3 TCID50 SCV2. At 3 or 7d post SCV2, lungs were harvested and processed for scRNA-seq (*n*=pool of 3-4 mice/group), flow cytometry or multiplex cytokine assay (*n*=7-15 mice/group; 2-3 independent experiments). Statistical significance was assessed using a linear mixed-effects model with pairwise comparison using JMP software. (**A**) UMAP of all cells separated by experimental group. (**B**) Heat map depicting cluster defining genes used for cell type calling in Fig4A. (**C**) Proportional cellular composition based on scRNA-seq clusters defined in Fig4A. (**D**) Feature plot showing expression of characteristic alveolar macrophage genes, *Marco*, *Mrc1*, *Chil3*, *Car4*, *Ear1*, *Ear2*, *Plet1*, *Fabp1*, *Fabp4* in re-clustered monocyte-macrophage compartment from scRNA-seq data at d0 (Fig3A) and 7dpi SCV2 (Fig4A) (**E**) Frequency of $TNF\alpha^+$ $CD8^+$ T cells as determined by flow cytometry. (**F**) Differential expression analysis of CD8+ T cells from *Nb* and control samples showing the top 50 DEGs. Enriched pathways are listed on the lefthand side. Each column represents an individual cell. (**G-H**) Protein levels of TNFα (**G**) and IFNγ (**H**) at 3d and 7d post SCV2 as measured in whole lung homogenate by multiplex cytokine assay. (**I**) Granzyme B expression by SCV2 spike-specific CD8+ T cells. (**J-L**) K18-hACE2 mice were inoculated with 500 *Nb* larvae by s.c. injection at d-28. Mice were then treated with either clodronate liposomes or control liposomes by i.n. and/or i.v. administration from d-6 to d-1 prior to SCV2 challenge on d0. Lungs were harvested at 7dpi. *n*=6-19 mice/group; 2 independent experiments. Statistical significance was determined by Kruskal-Wallis test with Dunn's post-test using the control liposome group as the fixed comparator. (**J**) Frequency of lung CD8+ T cells as determined by flow cytometry, separated by treatment group. Data are mean ± SEM. (**K-L**) Viral loads measured by qPCR (**K**) and TCID50 assay (**L**), separated by treatment group. Geometric mean is shown. Dark gray box indicates values below limit of detection. ns $p>0.05$; * $p<0.05$; ** $p<0.01$; *** $p<0.001$; **** $p<0.0001$
